# Insight Into the Role of Protestant Christianity in the Experience of Living With a Suicidal Relative: A Constructivist Grounded Theory Study

**DOI:** 10.1111/jpm.70025

**Published:** 2025-08-21

**Authors:** Christina Hennipman‐Herweijer, Joke van Nieuw Amerongen‐Meeuse, Janneke de Man‐van Ginkel, Nynke Boonstra, Hanneke Schaap‐Jonker

**Affiliations:** ^1^ Nursing Science in Mental Health Care UMC Utrecht Utrecht the Netherlands; ^2^ Center for Research and Innovation in Christian Mental Health Care Eleos/de Hoop Hoevelaken the Netherlands; ^3^ Eleos Mental Health Care Bosch en Duin the Netherlands; ^4^ Nursing Sciences, Program in Clinical Health Sciences Utrecht University Utrecht the Netherlands; ^5^ Faculty of Religion and Theology Vrije Universiteit Amsterdam the Netherlands; ^6^ Academic Nursing, Department of Gerontology and Geriatrics Leiden University Medical Centre Leiden the Netherlands; ^7^ NHL Stenden University of Applied Science Leeuwarden the Netherlands; ^8^ KieN Early Intervention Service Leeuwarden the Netherlands

**Keywords:** caregivers, family, qualitative research, religion, suicide

## Abstract

**Introduction:**

Living with a suicidal relative impacts multiple life aspects. However, it is not known how religious beliefs and meaning‐making influence relatives' experiences.

**Aim:**

This study aimed to develop a theoretical framework to understand the role of religion in the experiences of Christian relatives living with a suicidal loved one.

**Methods:**

A constructivist grounded theory study was conducted, adhering to the COREQ checklist. Fifteen interviews were conducted with seventeen Christian relatives of suicidal individuals.

**Results:**

Four themes emerged—acceptance of suicidality, seeking and experiencing God's help, surrendering to God, and religion's influence on relationships—forming a framework on how relatives' religious convictions about suicide and the intensity of their personal relationship with God influenced to what extent religion was helpful or harmful.

**Discussion:**

Christian faith provided peace of mind and support to relatives who had a personal relationship with God and believed their loved one would go to heaven. Relatives who believed their loved one would go to hell due to suicide and lacked a relationship with God experienced guilt and fear, making them vulnerable to harm from religion.

**Implications for Practice:**

Educational institutions and policymakers should specifically empower nurses to discuss spiritual issues when supporting relatives of suicidal individuals.

**Relevance Statement:**

This study offers valuable insight into the role of religious beliefs and meaning‐making influencing the experiences of Christian relatives living with a suicidal loved one. It highlights religion‘s role in their coping processes. The findings equip mental health nurses with a deeper understanding of religious coping strategies, enabling them to provide better support to relatives. Integrating spirituality/religion into support allows nurses to respond more holistically to relatives‘ needs, potentially improving their well‐being and resilience. Spiritual care is a part of nursing, but nurses feel unprepared. It is recommended that education and policy focus on nurses to better support relatives.

**Summary**

- Originality (what this paper adds to existing knowledge)
  ○ A framework was developed showing how relatives' religious convictions about suicide and the extent of their personal relationship with God determined whether religion was helpful or harmful.
  ○ Religion offered peace of mind and support to relatives with a personal relationship with God who believed their loved one would go to heaven. Conversely, those who believed their loved one would go to hell and lacked this personal relationship experienced guilt, fear, and potential harm from religion.
  ○ Being faithful in relationships was an important value
  ○ Openness about suicidality determined the extent to which needs were met within the relationship.
- Significance (what are the implications or potential impacts of the paper)
  ○ Nurses, due to their approachability and availability, are well‐positioned to identify spiritual needs of relatives and provide the necessary support. Education should focus on training nurses to recognise and guide relatives' religious struggles.
  ○ Policymakers may do well to support nurses in integrating spirituality/religion into counselling for families of suicidal individuals, as this could enhance support during critical religious and emotional challenges; for example, by updating guidelines and fostering collaboration with religious organisations.
- Rigour (what approach was taken to ensure the method used was good quality)
  ○ In‐depth, unstructured interviews conducted by a researcher with clinical expertise and religious sensitivity ensured rich data collection. Data saturation was confirmed by an additional interview to ensure comprehensive exploration of the research question.
  ○ Reflexivity was maintained through acknowledging preconceptions, memo‐writing, and discussions with other researchers, minimising bias and enhancing credibility. Constant comparison and collaborative analysis with a second researcher ensured analytical depth and consistency.
  ○ The co‐construction of meaning between the researcher and participants facilitated a nuanced understanding of their experiences, grounding theoretical constructs in participants' narratives and enhancing the credibility of the findings.

## 1 Introduction

Every year 703,000 people die by suicide worldwide, and even more experience suicidality (World Health Organization 2023). Suicidality is defined as having persistent thoughts about intentionally ending one's life (suicidal ideation) or a purposeful act of self‐injury where the intent is death (suicidal behaviour) (Nock et al. 2008; Clayden et al. 2012).

Approximately 11% of the general population reported being exposed to a suicide attempt by a family member (Hvidkjær et al. 2021). Although many family members have not chosen the role of caregiver, they are willing to contribute to the survival of their relative (Sellin et al. 2017; Wayland et al. 2021). However, caring for a suicidal loved one is a heavy burden that affects many areas of life: it adversely affects well‐being, puts pressure on family relationships, and managing the risk of suicide is difficult (Hennipman‐Herweijer et al. 2024). Often, relatives experience a lack of collaboration and acknowledgement from healthcare professionals and others, making them feel lonely (Vandewalle et al. 2021). Nevertheless, family support and openness about suicidality have been shown to be protective factors in suicide prevention (Frey et al. 2016). Relatives can make a valuable contribution to treatment and prevention (van de Bovenkamp and Trappenburg 2010).

Religion, defined as “a search for significance in ways related to the sacred” (Pargament 1997, 32), is used by many people to give meaning to events and experiences, and serves as a source of support in coping processes (Park 2013). While religion can provide support, it may also impose moral judgements on suicidality. Christianity has a long history of condemning suicide, dating back to Augustine (354–430) (Potter 2021). Many Christians still regard suicide as a sin; some see it as a mortal sin leading to damnation, while others consider it to be a sin equal to all others (Potter 2021). This view may lead to conflicts and hinder from seeking help (Binnix et al. 2018; Moksony and Hegedűs 2021).

Religion is one form of experiencing and expressing spirituality (Charzyńska and Heszen‐Celińska 2020). Addressing spirituality is a recognised part of holistic nursing care and is valued by patients and families, particularly in times of crisis (Zumstein‐Shaha 2021; Johnston Taylor et al. 2023). According to Greasly et al., spiritual care in mental health nursing “relates to the acknowledgement of a person's sense of meaning and purpose to life which may, or may not, be expressed through formal religious beliefs and practices” (Greasley et al. 2001, 636). The NANDA International Nursing Diagnoses Classification, the Nursing Interventions Classification (NIC) and the Nursing Outcomes Classification (NOC) offer nurses frameworks for addressing existential themes, including spirituality and religion (Cavendish et al. 2003; Moorhead et al. 2018; Bulechek et al. 2020; Herdman et al. 2024). However, mental health nurses face barriers when responding to patients' and families' spiritual needs. They often feel unprepared to provide spiritual care, and have difficulty distinguishing between spiritual needs and psychological symptoms (Elliott et al. 2020; Neathery et al. 2020).

Although it is known that having a suicidal loved one affects many areas of life (Lavers et al. 2022; Hennipman‐Herweijer et al. 2024), and that religion—particularly moral objections toward suicide and fear of hell—serves as a protective factor for suicidal persons (van den Brink et al. 2018), it is unknown how religious relatives experience living with a suicidal loved one. While it is likely that religion influences this experience due to the search for significance or moral objections, research is lacking on how religious beliefs and meaning‐making affect relatives' experiences. Therefore, a deeper understanding is required to appropriately comprehend the experiences of relatives, support them in their coping process, and contribute to their spiritual well‐being. To achieve this, the present study aims to develop a theoretical framework for understanding the role of religion in the experiences of Christian relatives living with a suicidal loved one.

## 2 Methods

### 2.1 Design

This study was conducted between February 2022 and June 2023 and used a constructivist grounded theory (CGT) methodology (Charmaz 2006). As there is little known about the role of religion in the experiences of family members of suicidal individuals, developing a theory can enhance understanding. The relativist epistemology of CGT aligns with the dynamic perspectives on suicidality over time. The CGT research process emerges from the interaction between the researcher and the participant (Charmaz 2008, 2017). It acknowledges the subjective position and encourages the use of the experience and expertise of the researcher and the participants, each with their own interpretations of their experiences.

The COREQ guidelines were followed (Tong et al. 2007).

### 2.2 Participants

The target population comprised Christian relatives of suicidal persons receiving treatment in mental healthcare. To be included in the study, the participant had to have a close relationship (e.g., partners, offspring, siblings, close friends) with a person who was suicidal in the past year and is still alive. Additionally, participants needed to be older than 18 years, speak fluent Dutch and, regardless of their relatives' religious beliefs, consider themselves Christian for whom faith is an essential part of their lives. Since no previous research has been performed into the experiences of religious relatives of suicidal individuals, this study explored this topic among Christian relatives because Christianity is the largest religion in the Netherlands (Pew Research Centre 2012).

Purposive sampling was conducted to obtain maximum variation in the type of underlying DSM‐5 classification of the suicidal person, relationship type, time and duration of living with them, and religious affiliation of the relative and the suicidal person. Recruitment was done by therapists not involved in the study at two Dutch mental healthcare organisations, one of which was a Christian organisation, and included both inpatient and outpatient patients. Patients who were suicidal and undergoing treatment in the past year received a letter with information about the study, which they could give to a relative important to them. Based on the relatives preferences, they subsequently contacted or were contacted by the researcher. Patients were assured that inviting relatives was non‐compulsory and did not affect their treatment.

Seven men and ten women, aged between 19 and 78, participated in the study. Although Catholic Christians were not excluded from the study, only Protestant Christians participated. These Protestant Christians can be divided into three groups: (1) Pietistic Reformed: Emphasise personal conversion, sanctification, and emotional faith while strictly adhering to Calvinist doctrines, including Scripture's infallibility and predestination; (2) Orthodox Reformed: Emphasising biblical interpretation, covenant, and conservative Protestant theology while upholding historic creeds and traditions; (3) Evangelical: Focus on personal conversion, biblical authority and an active missionary approach, often with an emphasis on social engagement and contemporary relevance of faith. In two of the interviews, both parents of the suicidal person participated. One participant came from a third organisation and was eager to participate in the study. This participant was included after applying the inclusion criteria and maximum variations criteria. For more details, see Table 1.

**TABLE 1:** Socio‐demographic details about participants and their relatives.

Characteristics	Number	Percentage
*Characteristics of participants* (*n* = 17)		
Gender		
Male	7	41
Age (19–78 years, Mean: 49)		
19–29	2	12
30–39	1	6
40–49	7	41
50–59	3	18
60–69	1	6
≥ 70	3	18
Relationship		
Spouse	6	35
Parent	6	35
Offspring	2	12
Sibling	3	18
Living together	8	47
Last time family member was confronted with relative's suicidality		
Less than a week	4	24
Less than 3 months	8	47
3 to 6 months	4	24
9 to 12 months	1	6
Religious affiliation		
Pietistic Reformed	6	35
Orthodox Reformed	7	41
Evangelical	4	24
*Characteristics of suicidal relatives* (*n* = 15)		
Gender		
Male	7	47
Age (18–80 years, Mean: 43)		
18–29	5	33
40–49	4	24
50–59	4	24
60–69	1	7
≥ 70	1	7
Distribution by mental health care organisation		
A	10	67
B	4	27
C	1	7
Diagnosis according to participants		
Unknown	3	20
Depression	3	20
PTSD	1	7
Depression and autism	1	7
Depression and personality disorder	1	7
Depression, PTSD and personality disorder	1	7
Bipolar disorder	2	13
Autism and addiction	1	7
Asperger syndrome	1	7
Dissociative identity disorder	1	7
Number of known admissions to a psychiatric hospital		
Never	1	7
Once	2	13
Twice	2	13
More than twice	7	47
Number of known suicide attempts		
Never	3	20
Once	3	20
Twice	1	7
Thrice	2	13
More than 3 times	4	24
Yes, but number unknown	1	7
Christian	12	80

### 2.3 Ethical Considerations

The study was approved by the ethics committees of the participating mental health organisations, as reported by the committee secretaries by email on 2022/02/02 and 2022/02/08. Data were handled confidentially and were pseudonymised. After receiving both written and oral information about the study, all participants gave their written consent before the interview. Interviews ended with debriefing questions; if the interview evoked distress, contact with a therapist was offered.

### 2.4 Data Collection

In‐depth interviews were conducted to co‐create meaning with participants by reconstructing experiences (DiCicco‐Bloom and Crabtree 2006). This open, unstructured form of interviewing provides detailed descriptions of experiences (Vandermause and Fleming 2011). All interviews were conducted by CHH, a Christian mental health care nurse working in a ward where suicidality is common, with a focus on the family of patients. This researcher, who has a pre‐understanding of the issue due to her professional background, sees suicide as contrary to God's will, but as a result of illness that deserves compassion rather than judgement. According to Gadamer, understanding is only possible with historical awareness; therefore, pre‐understanding was not seen as prejudice (Gadamer 1993). Hence, bracketing is defined as an attitude of persistent curiosity in which the researcher actively avoids seeking confirmation of existing theories or expectations, instead maintaining a flexible and open stance that remains receptive to new insights and perspectives (LeVasseur 2003). Throughout the interview, the interviewer progressively delved deeper into participants' experiences driven by a sense of curiosity, thereby facilitating the emergence of the meaning attributed to these experiences. The researcher's prior knowledge had a positive impact on participants' understanding because of her awareness of sensitive topics (Vandermause and Fleming 2011). The researcher avoided steering the conversation and ensured that the participant's story was central (LeVasseur 2003). Interviews were also discussed with non‐religious researchers to avoid making assumptions.

After developing rapport, three questions reflecting the core topics of this study were asked: “Can you tell me about what it is like for you to have a suicidal relative?”, “How do your religious beliefs influence your experiences?”, and “How do you give meaning to this situation and what role does your religion play in it?” A pilot interview was conducted by CHH and reviewed by HSJ to assess whether the core topics were useful. These questions remained unaltered, as they both provided guidance and room for open discussion, allowing pertinent themes to surface naturally during data collection. The questions did not determine the structure of the interview but served as a reminder of the subject of study. Member checking was performed by providing a summary at the end of the interview and asking whether this summary corresponded to the participant's experience. After each interview, field notes were taken regarding the atmosphere, unspoken emotions, and non‐verbal communication.

Fifteen interviews were conducted, lasting between 44 and 116 min (mean duration: 80 min). The interviews were carried out at the participant's preferred location (home *n* = 11, or the treatment location of their relative *n* = 4) and were audio‐recorded and transcribed. After data saturation was achieved, as determined by the constant comparison method by analysing the data after each interview, one additional interview was conducted to confirm the findings.

### 2.5 Data Analysis

The data analysis followed Charmaz's (2006) methodology. The analysis was performed by the researcher (C.H.‐H.). The first interview was also analysed by a second researcher (J.N.A.‐M.), a physician with clinical experience with suicidality and expertise in qualitative research. The generated codes and categories were compared; any differences were resolved by consensus. For later interviews, any uncertainties were discussed with J.N.A.‐M. or H.S.‐J. until a consensus was reached.

Initially, open line‐by‐line coding was conducted, staying close to participants' expressions to remain open to various theoretical possibilities. Categories were developed using constant comparison, reviewing earlier interviews for consistency. Memos documented emerging ideas and patterns both within and across the interviews.

During the focused coding phase, codes and categories were refined and organised. A code tree was developed for individual interviews and the overall dataset, with each part informing the whole and vice versa, as the main themes permeated the entire text (Manen 1997). Relationships between categories were established by examining context, causality, and consequences, linking them to the central themes that emerged. The identification of patterns and subsequent development of the final code tree were conducted by C.H.‐H., J.N.A.‐M., and H.S.‐J. The theory was developed by integrating the intersecting categories (Creswell and Poth 2018, 84).

Given the interpretive nature of CGT, the researcher adopted a reflexive stance during the analysis by acknowledging her own standpoint throughout the analysis and keeping memos (Fleming et al. 2003). Preconceptions were made explicit through discussions with J.N.A.‐M., H.S.‐J. and J.M.‐v.G. to allow prior knowledge to aid in data analysis and the interpretation of meanings (Manen 1997).

MS Word was used to manage the data from the interviews; each step of the analysis was stored as a separate step.

## 3 Results

Relatives reported two moral views about suicidality. The first was the belief that suicide is a sin leading to hell. The second was the belief that suicide, like all other suffering, is not God's intention but entered the world as a consequence of sin. Consequently, some relatives viewed it as a sin that God can forgive, while others perceived it as a consequence of illness brought into the world by sin and therefore not subject to personal condemnation.

These views affect four themes that emerged from the data: Acceptance of suicidality is determined by the expected hereafter of their relative, seeking and experiencing God's help, surrendering to God, and religion influencing relationships toward others.

### 3.1 Acceptance of Suicidality Is Determined by the Expected Hereafter of Their Relative

The place in the hereafter appeared to be more important to relatives than the death by suicide itself. If a relative was convinced that their loved one would go to heaven, suicide was accepted. In that case, the desire for their loved one's suffering to end was stronger than their conviction that suicide is contrary to God's intention or grief over loss.

I'm totally fine with it, if it happens, yeah. (…) She really suffers. (…) I keep thinking about: the way she's living, it's just inhumane. (Parent)

The expectation that their loved one will go to hell derived from the conviction that committing suicide is an unforgivable sin or from their relative's lack of belief in God. Fear that their loved one will go to hell made it impossible to accept suicide.

He goes: “(…) Do you honestly believe that (…) if I keel over right now, I'm headed straight for hell?” So I reply, “Well, that's not my call. But if you're not living with God, I can't take solace in the thought that you're coming home (in heaven).” (Parent)

In an attempt to change the situation, relatives appealed to their loved ones' moral stance on suicide and stress the importance of coming to faith. Their fear of hell was discernible even in their choice of terminology, with relatives preferring to speak “not going to heaven” rather than explicitly stating “going to hell”.

Preventing suicide could have different connotations depending on the expected hereafter. If the loved one was expected to go to heaven, relatives did not want to facilitate an attempt, acting out of a sense of responsibility. If hell was the expected hereafter, relatives felt compelled to prevent their loved one from going there, acting out of fear.

But I also always said, “(…) If I can save you (…) I won't let you die. That would be like committing murder.” And I say, “I'm not killing you. I'm not gonna even to allow you to have that possibility that you can do that”. (Parent)

### 3.2 Seeking and Experiencing God's Help

Most relatives brought their concerns to God in prayer when they felt powerless. Prayers were offered for the soul's preservation, protection from suicide and recovery for their loved one, and wisdom and health for oneself. If support was expected from God, it was usually experienced. These relatives usually engaged in regular interaction with God throughout the day and maintained a profound trust in His constant presence. Frequently, relatives reported a sense of restfulness following their prayers.

Then I put it to God, “Hey, do You want to be there? I'm not sure how it'll all play out, but I do know You're around.” And then, yeah, it kinda brings a bit of peace or something. (Spouse)

The Bible served as a source of God's answers and support, particularly through stories where recognition and guidance were found, with the stories of Moses and Job being the most frequently mentioned. Moreover, many of the intense emotions that relatives experienced were recognised in the Psalms, which revealed to them who God is and provided guidance in life. In addition, relatives also received support and answers through sermons, hymns, religious apps, and other people. Beyond moral support, God's help was experienced practically.

The fact that they could bear it with God instead of alone provided them strength and stability. Even if their prayers remained unanswered, this enabled them to accept that God provides in a different way than requested.

And I actually wanted a response from God. And God's answer to Job is not really of any use for us (…) saying that all these problems came into the world through the Fall. (…) I can't do anything with that, (…) but it provides a connection with God. (Spouse)

Relatives who considered God as holy and all‐powerful while seeing themselves as disenfranchised expected less from prayer. As a result, they rarely requested help from God or implicitly expressed their needs, and they seldom experienced support from God.

Well (I miss) a little bit of God's care, you know. Like, on those days when things just don't go right, I'm like, “Where's that care at?” But, of course, you can't really say that. (Parent)

The absence of a sense of God's presence was perceived as difficult. This resulted in feelings of loneliness and frustration, with the latter occasionally transforming into a sense of resistance.

There are people who say, “I prayed to God and got some help.” (…) Then I think: “yeah, I don't recognize that”. (…) It makes me wonder, you know, “Do they have some special connection, or what's the deal? (…) Am I just not seeing it enough?” That is certainly difficult for me. (Spouse)

Generally, during a crisis, the first impulse was to act to gain control of the situation rather than asking God for help. When God's help was requested immediately afterwards, strength was rapidly experienced again, leading to a deepening of faith. Some relatives who felt guilty about not involving God confessed their sin but did not experience forgiveness. This created distance from God.

Well, then you end up feeling even guiltier because you didn't turn to the Lord for help. (Offspring)

### 3.3 Surrendering to God

Relatives placed their trust in God within their circumstances due to His divine nature and engagement, His overarching plan, and His providence. Many relatives trusted in the hope that better times will follow in eternal life and that this suffering is only temporary.

So, that's my fundamental belief, and it doesn't hinge on how much I pray, how often I express gratitude, or how much I dive into the Bible. It's rooted in the fact that God is trustworthy. (Sibling)

All relatives believed in God orchestrating life and meeting needs. As this collective conviction became more personal, trust deepened. Those with strong trust in God felt that regardless of outcomes, it would be fine because God would take care of them. This trust led to an active surrender to God. This included the choice of continuing to trust if a subsequent suicide attempt succeeds.

Of course you know there is One who rules and governs everything and you can go there with everything, but you also know, it's not always fulfilled. (…) And sometimes that is disappointing (…). But still, at a certain moment it is: “Thy will be done”. (Parent)

Surrendering relatives recounted various experiences about how relief came in unexpected ways during difficult times. This strengthened their confidence that God is in control, giving them peace of mind.

But somewhere there is a kind of peace, than I think: “whatever happens, it's not my fault.” (Sibling)

However, there appeared to be a tension between complete surrender to God and personal responsibility driven by the conviction that God has placed them in this position for a reason; feeling responsible for preventing suicide. The conviction that they could share the responsibility with God helped them bear this.

Nearly all relatives struggled with God's plan at some point. When this anger was addressed to God out of a desire to restore the relationship with God, surrender almost always followed.

I must try to come out of this (…). I was angry at God and I had to do something with that. (Spouse)

Relatives who lived by the conviction that God controls everything, but did not experience God's involvement personally, did not come to surrender, and noticed little change in their own faith, which fits their reported view on God as distant and sovereign.

I don't know if I can say it that way, but He makes His Own choices of course. (Sibling)

When relatives did not talk about surrender, they usually reported guilt for questioning God's omniscience. Expressing the conviction that God is in control, without internalising it, caused struggle rather than rest. In such cases, relatives doubted whether they would preserve their faith and receive strength from God if suicide were to occur.

On one hand, you know that the Lord doesn't owe us any explanations, but still you would want to hold Him accountable, and at the same time, you hesitate to do so. (Offspring)

Relatives' reported that their decision to leave judgement to God was also liberating. This was particularly evident among relatives with non‐believing loved ones. Surrendering judgement to God was also present among relatives whose conviction was that their loved one would be going to hell because of suicide. This belief caused tension, as their firm conviction did not allow for a different judgement by God.

That's a false hope yeah, but because it's your own child then it's soon anyway, then you start talking it in the right direction, but you shouldn't do that either. (Parent)

### 3.4 Religion Influencing Relationships Toward Others

First, religion influenced the relationship with the suicidal person. Despite the changes in and pressures on relationships in these circumstances, being faithful was seen as an essential value. Notwithstanding the pressures on marriage, spouses chose to honour their wedding vows.

We have been married for thirty years this year. I say: “That's something I just don't think about (divorce). That door is just locked for me.” (…) Yes it is for better or for worse. So also this, no matter how tough it is. (Spouse)

Despite considerable involvement with their suicidal loved one and seemingly strong relationships, there was hardly any openness about suicidality, especially in families where suicide was condemned.

No, my mother never talks about that either. Not even with my siblings. (Offspring)

Second, relatives experienced both help and struggles in their religious network. When relatives wanted to share their concerns, they sometimes seek out individuals within their religious network. Some requested fellow Christians to pray for them. This prayer was seen as valuable and supportive.

I just know prayer is got my back, you know? (…) When [partner] was heading for the hospital, I made sure there was someone at home every day, keeping him company. (…) And at the same time, I can bring up my prayer requests or things I'm thankful for. (Spouse)

The congregation regularly extended assistance and compassion through various means, such as lending a receptive ear, sending cards, providing practical support, or engaging in intercessory prayer. Nevertheless, numerous relatives encountered stigma related to suicidality within the congregation. In an effort to prevent their loved one from being perceived differently or facing condemnation, they seldom disclosed their circumstances to others. In some cases, judgement was passed from the church congregation.

Because of course I am very often told that it is sinful, and then you are lost, it's often stated so unequivocally. (Parent)

Similar to relatives, church members and pastors, also exhibit a reluctance to discuss suicide. Specifically, the reluctance of pastoral team members was viewed as burdensome, as numerous relatives sought spiritual guidance to address their concerns.

Many pastors are very shy when it comes to mental health issues. Then they don't know. (…) (That's) frustrating. And you do have questions yes. And that, I just don't get, for my feeling then, any answers to that. (Spouse)

### 3.5 Theoretical Framework

**FIGURE 1:**
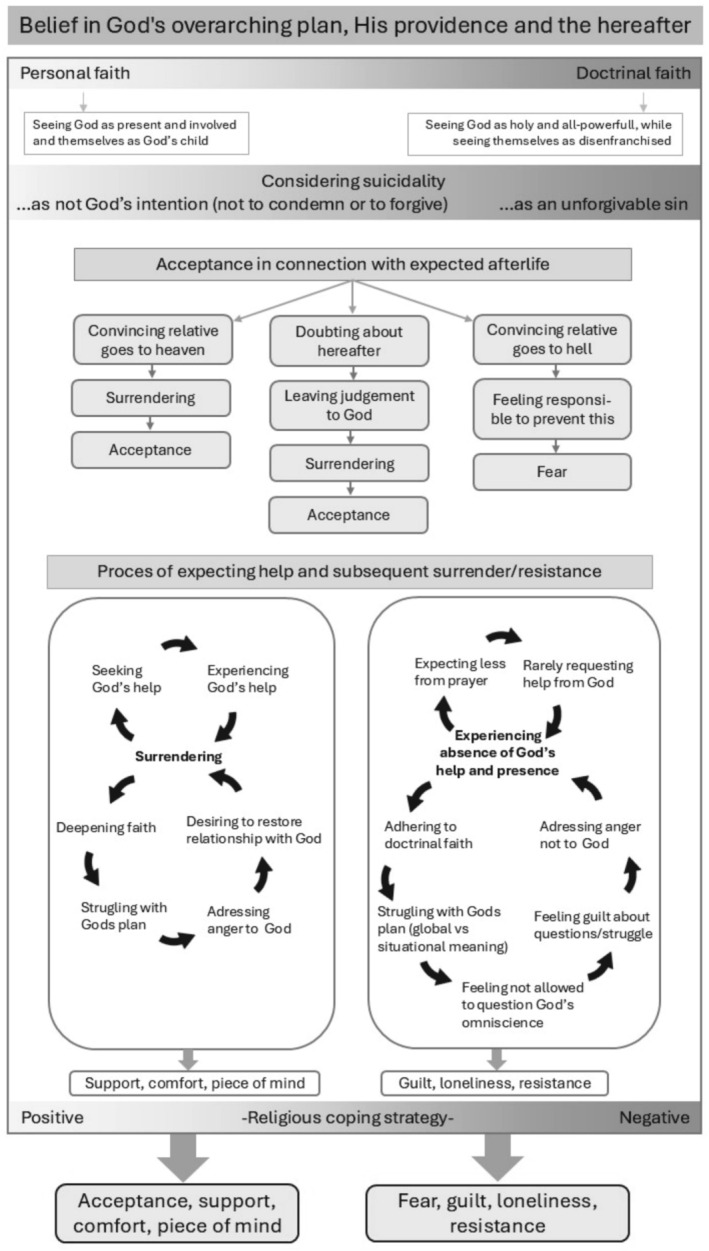
Theoretical framework for the role of religion in the experience of relatives living with a suicidal loved one in the vertical relationship. Three continuums influence the outcomes (gradients), with elements on the left corresponding to lighter colours and those on the right to darker colours.

**FIGURE 2:**
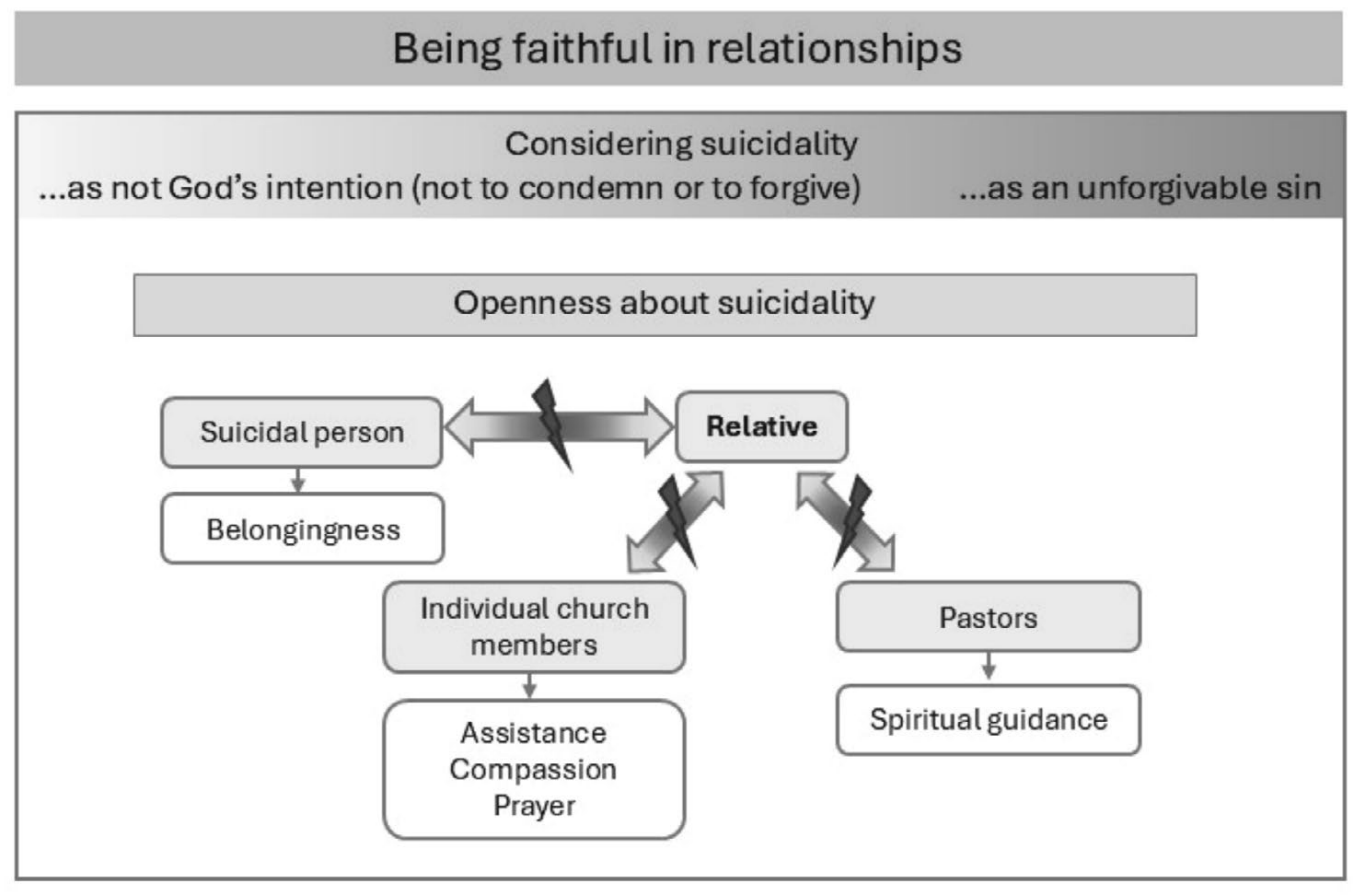
Theoretical framework for the role of religion in the experience of relatives living with a suicidal loved one in horizontal relationships. One continuum is influential, with disruptions in contact primarily associated with darker colours, represented by lightning symbols. Stigma operates in both directions. The white blocks indicate what the contact yields, but also what is missed when disruptions occur.

A belief in God's plan, providence, and the afterlife formed a common foundation among all participants. Differences were represented along a continuum. The first continuum contrasted personal belief in God's involvement with doctrinal faith based on religious teachings. The second addressed views on suicidality, seeing it either as not part of God's intention and therefore not condemnable or forgivable, or as an unforgivable sin. The third focused on religious coping strategies that emerged during the process. Acceptance, closely tied to the afterlife, emerged as a key theme, with surrender to God as a central theme. Those on the darker end struggled with surrender, while those on the lighter end linked surrender to acceptance. Seeking and experiencing God, along with surrender, were mutually reinforcing, leading to peace of mind. Most participants were at the extremes, distinguishing those who felt peace and acceptance from those who experienced fear, guilt, loneliness, and resistance (Figure 1). All participants applied the religious value of being faithful in relationships. Stigma around suicidality hindered openness from all involved, which affected the fulfilment of needs within relationships. Although consideration on suicidality seemed to affect this, it was less clear than for other themes (Figure 2).

## 4 Discussion

Religion had both a positive and a negative role in the experience of Christian relatives living with a suicidal loved one. Religion provided comfort and support when relatives asked for God's help, surrendered to God out of trust in Him, and when there was the conviction that their relative would go to heaven; this led to acceptance and peace of mind. In contrast, if relatives believed that suicide leads to hell and had no personal relationship with God, feelings of fear and guilt arose, along with a perceived lack of support from God.

Fundamental to all participants was their use of religion to cope with this stressful circumstance. Many relatives used positive religious coping strategies, as described by Pargament et al. (1998): seeking spiritual support, collaborating with God, benevolent religious reappraisal, religious focus, and religious surrender. In particular, surrendering to God and letting go of control accounted for a large proportion of peace of mind. This is consistent with previous research showing that surrender is negatively correlated with stress and positively with spiritual well‐being (Wong‐Mcdonald 2000; Clements and Ermakova 2012). In contrast, the group that used negative religious coping strategies (Pargament et al. 1998), such as spiritual discontent and reappraisal of God's powers, experienced more difficulty with the situation. This led to fear, guilt, and a perceived distance from God. This aligns with a study of caregiver burden in family members of stroke patients, which found that caregiver burden was negatively correlated with positive religious coping and positively with negative religious coping (Kes and Aydin Yildirim 2020). Pargament and Exline (2021) argue that the use of positive religious coping strategies is a predictor of adjustment to life stress.

Relatives holding the religious belief that suicide leads to hell faced significant challenges. This aligns with Park's meaning‐making model, where stress arises from a disparity between global meaning (individuals' general orientation system) and situational meaning (individuals' interpretation of specific situations); (Park and Folkman 1997; Park 2013). In the current study, relatives found the judgement harsh, condemning the behaviour but not the person. They wished for salvation but struggled to reconcile it with their global meaning, causing stress. This stress eased when their global meaning shifted through experience, especially among those using positive religious coping strategies (Park 2013).

Anger toward God emerged in different variations among almost all participants. For those with a close relationship with God, anger was not avoided, but contact with God was sought, leading to a deepening of the relationship. This is complementary to research stating that a clear commitment to preserve religious faith as part of a close relationship with God gives room for questioning and complaining (Exline et al. 2012).

Despite the notion that churches are rich sources of social support, the current study reveals that, akin to findings in other research, religious relatives might feel isolated from support (Krause et al. 2001; Spillane et al. 2019). The reticence to discuss suicidality is unfortunate, since spiritual support from church members promotes the use of positive religious coping styles, leading to a lower caregiver burden (Wong‐Mcdonald 2000; Krause et al. 2001). This theme, primarily focusing on the relationship with fellow Christians, demonstrated minimal interaction with other themes, primarily focusing on the relationship with God. This may be because among the Protestant Christians of our sample, the relationship with God is mainly seen as vertical, while relationships with others are considered horizontal (Goodenough 2001; Streib and Hood 2013). With some caution, it can be suggested that those who condemn suicide may experience loneliness in both vertical and horizontal relationships.

While this study bears similarities to previous studies, there are also differences. A sense of responsibility is a factor of burden in other studies (Nosek 2008; Buus et al. 2014), but plays a lesser role here, possibly because some of the responsibility is shared with God. Additionally, previous qualitative research suggests that strained relationships lead to rejection and divorce (Buus et al. 2014; Nygaard et al. 2019), but the current study shows that relatives are faithful in relationships. This may be due to compassion and faithfulness, which are important guiding values for believers (Park and Hale 2014).

The role of religion for relatives in the context of suicidality has not been investigated before. A strength of the current study is that participants, who varied demographically, were eager to share their stories with the interviewer. The shared Christian background and understanding of living with a suicidal loved one fostered trust and collaboration. In addition to enhancing reliability through collaborative analysis with another researcher, this approach also facilitated the explication of emotions that emerged during interviews and analysis. This was particularly necessary after interviews with relatives who believed their loved one was destined for hell, as their pain and helplessness felt profound.

### 4.1 Limitations and Recommendations

The sample was demographically diverse, but only Protestant Christians participated, making the findings not generalisable to other religious backgrounds. Although the four major world religions—Christianity, Islam, Hinduism, Buddhism, and Judaism—are all protective against suicide and mostly regard suicide similarly, differences exist (Saiz et al. 2021). Therefore, more research is needed on other religions, particularly regarding how a lack of openness affects the relationship between religious families and the suicidal relative and the relationship between religious families and their network. Qualitative research can provide insight into the impact of stigma on mutual relationships, how the religious community can support relatives, and how families deal with reactions from their religious community.

## 5 Relevance to Clinical Practice

Due to their approachability and availability for ad hoc conversations, nurses are well‐suited to guide relatives in their meaning‐making process and are the preferred professionals for discussing spiritual issues (van Nieuw Amerongen‐Meeuse et al. 2019). As nurses generally consider spiritual care an integral part of their practice but do not always feel adequately prepared for this role, it is recommended that education and policies focus on supporting nurses. Educational institutions would do well to pay more attention to recognising religious coping strategies and guiding relatives with religious struggles by providing training on how professionals can (a) be trusted partners, (b) be present during religious struggles, (c) identify and normalise religious struggles, (d) facilitate acceptance and reflection, and (e) assist in accessing resources (Pargament and Exline 2021). These elements align closely with several nursing interventions described in the Nursing Interventions Classification (NIC), namely: (a) Complex Relationship Building and Counselling, (b) Presence, (c) Spiritual Support and Emotional Support, (d) Coping Enhancement and Hope Inspiration, (e) Religious Ritual Enhancement, and Spiritual Growth Facilitation (Bulechek et al. 2020).

## 6 Conclusion

This study of the experiences of Christian relatives living with a suicidal loved one shows that religion can be both helpful and harmful. This investigation also illuminates the significance of positive religious coping strategies, such as surrender and collaboration with God, facilitating peace of mind and reduced stress. This is contrasted with the use of negative religious coping styles, such as spiritual discontent, demonic reappraisal, and reappraisal of God's powers, which lead to fear and guilt. Despite the belief that church communities can offer valuable support during difficult times, relatives of suicidal individuals often lack this support, contributing to feelings of loneliness. Therefore, professionals, especially nurses, should be made aware of the religious convictions and coping strategies of relatives to provide appropriate support.

## Ethics Statement

All procedures performed in this study involving human participants were in accordance with the ethical standards of the institutional and/or national research committee and with the 1964 Declaration of Helsinki and its later amendments or comparable ethical standards. All participants provided informed consent before the interview for the audio recording and agreed to the use of this data for publication purposes.

## Conflicts of Interest

The authors declare no conflicts of interest.

## Data Availability

The data that support the findings of this study are available from the corresponding author upon reasonable request.
